# Profiling the Cardiovascular Toxicities of CDK4/6 Inhibitors: A Real-World Pharmacovigilance Study

**DOI:** 10.3390/cancers16162869

**Published:** 2024-08-17

**Authors:** Jae Hyun Kim

**Affiliations:** 1 School of Pharmacy and Institute of New Drug Development, Jeonbuk National University, Jeonju 54896, Republic of Korea; kimkimjh@jbnu.ac.kr; Tel.: +82-63-219-5638; 2 Biomedical Research Institute of Jeonbuk National University Hospital, Jeonju 54907, Republic of Korea

**Keywords:** CDK4/6 inhibitors, palbociclib, ribociclib, abemaciclib, cardiovascular toxicity, signal detection

## Abstract

**Simple Summary:**

CDK4/6 inhibitors are recommended as first-line therapy for treating patients with hormone receptor-positive metastatic breast cancer. The most commonly reported adverse events of CDK4/6 inhibitors include neutropenia, leukopenia, and diarrhea. Recent case reports and retrospective studies show that CDK4/6 inhibitors may be more frequently associated with cardiovascular adverse events. This study comprehensively analyzed the FDA Adverse Event Reporting System and provided the frequency and onset of cardiovascular adverse events, including heart failure, hypertension, myocardial infarction, QT prolongation, pericarditis, and cardiomyopathy. Hypertension and heart failure were frequently reported among the analyzed adverse events. Further research is warranted to investigate the underlying mechanisms of cardiovascular events.

**Abstract:**

Cyclin-dependent kinase 4 and 6 (CDK4/6) inhibitors are approved for the treatment of human epidermal growth factor receptor 2 (HER-2)-negative, hormone receptor-positive breast cancer. The cardiovascular toxicity of CDK4/6 inhibitors is not well understood. This study aims to profile the cardiac events associated with CDK4/6 inhibitors. Reports from 2015Q1 to 2024Q1 were obtained from the FDA Adverse Event Reporting System (FAERS). Reports identifying palbociclib, ribociclib, and abemaciclib as the primary suspect were examined for cardiovascular toxicity, including hypertension, cardiac failure, cardiomyopathy, arrhythmia, myocardial infarction, and myocarditis. Signal detection was performed using the proportional reporting ratio (PRR), reporting odds ratio (ROR), and information component (IC). A total of 69,139 reports were analyzed. The median time to adverse events was 69 days (interquartile range [IQR], 18–260 days). Of these, 2065 reports documented cardiac adverse events. Ribociclib and QT prolongation were re-confirmed as a signal (PRR 8.43, ROR 8.65, IC025 2.86). Hypertension and cardiac failure were the most frequently reported cardiovascular toxicities. This study demonstrates that the use of CDK4/6 inhibitors is associated with cardiovascular adverse events, such as heart failure and hypertension. Further research is needed to understand the mechanisms and risk factors contributing to the cardiovascular toxicity of CDK4/6 inhibitors.

## 1. Introduction

Breast cancer is the most prevalent cancer among females globally, accounting for nearly one in four cancer cases in women [1]. The incidence rates per 100,000 women vary by region, ranging from 26.7 to 100.3 [1]. Among the different breast cancer subtypes, the most common is human epidermal growth factor receptor 2 (HER-2)-negative, hormone receptor-positive breast cancer, which accounts for about 70% of all cases [2]. Treatment strategies for HER-2-negative and hormone receptor-positive breast cancer include aromatase inhibitors, cyclin-dependent kinase 4 and 6 (CDK4/6) inhibitors, fulvestrant, tamoxifen, and alpelisib [3,4,5].

Newer agents including CDK4/6 inhibitors have provided novel treatment options for HER-2-negative, hormone receptor-positive breast cancer. Ribociclib, palbociclib, and abemaciclib were approved in 2017, 2015, and 2017, respectively, and are used in combination with aromatase inhibitors or fulvestrant for the treatment of advanced or metastatic HER-2-negative and hormone receptor-positive breast cancer. Contemporary clinical guidelines recommend CDK4/6 inhibitors, including ribociclib, palbociclib, and abemaciclib, as part of the treatment regimen [3]. The regimens of aromatase inhibitor plus ribociclib, fulvestrant plus ribociclib, and fulvestrant plus abemaciclib have demonstrated overall survival benefits in phase 3 clinical trials and are currently recommended at the highest level in the National Comprehensive Cancer Network (NCCN) guideline [3].

The initial pivotal clinical trials conducted for CDK4/6 inhibitors, including PALOMA-2, MONALEESA-2, and MONARCH-3, have recently released their final analyses, including results for overall survival endpoints [6,7,8]. All three trials targeted patients with advanced breast cancer and co-administered CDK4/6 inhibitors with nonsteroidal aromatase inhibitors such as letrozole. The most commonly observed treatment-emergent adverse event in the clinical trials for palbociclib and ribociclib was neutropenia [7,8]. In the clinical trial for abemaciclib, the most frequently observed adverse event was diarrhea, followed by neutropenia and leukopenia [6]. Additionally, other reported adverse events include hepatotoxicity such as the elevation of aspartate aminotransferase (AST) or alanine aminotransferase (ALT), and pulmonary toxicity, including interstitial lung disease [9].

In addition to the adverse reactions identified in clinical trials, recent studies have reported cardiovascular toxicity associated with the use of CDK4/6 inhibitors [10,11,12,13]. These studies varied in design, including case reports and retrospective observational studies. The reported cardiovascular toxicities also varied, including drug-induced QT prolongation, hypertension, heart failure, myocardial infarction, and pericardial effusion [14,15]. When drugs are used in the real world, they are often administered for longer periods and to patients with different characteristics than those in clinical trials, potentially leading to adverse reactions that were not observed in clinical trials [16]. Additionally, the representativeness of the population in clinical trials may differ from that in the real world, which could lead to variations in the pattern of adverse events [17,18].

Understanding the cardiovascular toxicity caused by anticancer agents, such as CDK4/6 inhibitors, is of clinical importance. Profiling the cardiovascular toxicity associated with CDK4/6 inhibitors, including the type, onset, and frequency, is crucial for selecting the appropriate drug for patients with various cardiovascular risk factors. Therefore, this study aims to comprehensively screen for cardiovascular toxicity associated with the use of CDK4/6 inhibitors, evaluate the types of cardiovascular toxicity that are most frequently reported for each drug, and investigate the median time to onset of these toxicities, thereby providing new information not obtained in clinical trials.

## 2. Materials and Methods

### 2.1. FAERS Data Preparation

The FDA Adverse Event Reporting System (FAERS) was used to explore adverse effects associated with CDK4/6 inhibitors. The FAERS is a database containing spontaneous reports of adverse events and allows for the downloading of raw data files. FAERS raw data files from 2015Q1 to 2024Q1 were downloaded, considering that palbociclib was approved in 2015. Adverse event reports identified as erroneous by the FDA were excluded from the analysis. The workflow to create the analysis set for this study from the entire FAERS dataset is as follows: (1) download the raw dataset provided by the FAERS; (2) remove erroneous cases; (3) perform case deduplication; (4) exclude reports with unclear age information; (5) obtain a subset of adverse event reports with CDK4/6 inhibitors as the primary suspect.

The FAERS database includes multiple versions of the same adverse event case as initial and follow-up reports, which can result in duplicate reports for the same case. These duplicates were resolved by identifying *caseids* and retaining the most recent report. The FAERS database is known to have duplicate reports with different *caseids* [19]. To further remove these duplicate reports, cases where the age, event date, reporter country, adverse events, and prescribed drugs were identical were regarded as reports of the same event, even if the *caseids* were different, and were deduplicated accordingly. To select reports related to CDK4/6 inhibitors, we searched the *drugname* or *prod_ai* fields for each drug’s ingredient name or its variations, as well as each drug’s brand name or its variations.

### 2.2. Evaluation of Cardiovascular Toxicity

The International Cardio-Oncology Society has categorized the types of cardiovascular toxicity that can occur after the administration of anticancer therapies into cardiac dysfunction/heart failure, hypertension, arrhythmias/QT prolongation, myocarditis, and vascular toxicity [20]. For CDK4/6 inhibitors, various types of cardiovascular toxicity, ranging from QT prolongation to hypertension, have also been reported in recent reviews [14,15]. Therefore, we first aimed to identify the patterns of all adverse events related to cardiac disorders and subsequently perform signal detection for specific cardiac adverse events of interest.

FAERS data provide information on adverse events using the Medical Dictionary for Regulatory Activities (MedDRA) Preferred Terms. To identify adverse events in FAERS reports, the MedDRA version 27.0 was used. To identify reported cardiovascular toxicities, we first summarized the reporting frequency of all Preferred Terms under the System Organ Classification (SOC)-level term “Cardiac disorders” (SOC code = 10007541). Since individual reports might use different Preferred Terms with similar meanings to describe specific cardiovascular toxicities, we also utilized Standardized MedDRA Queries (SMQs) to comprehensively identify these reports. The SMQs used for cardiovascular toxicity included arrhythmia-related investigations, signs, and symptoms (SMQ code = 20000051), cardiac failure (SMQ code = 20000004), cardiomyopathy (SMQ code = 20000150), hypertension (SMQ code = 20000147), noninfectious myocarditis/pericarditis (SMQ code = 20000239), torsade de pointes/QT prolongation (SMQ code = 20000001), and myocardial infarction (SMQ code = 20000047). To increase specificity, searches using SMQs included only narrow terms [21]. The list of Preferred Terms included in each SMQ is provided in Supplementary Table S1.

### 2.3. Statistical Analysis

Descriptive statistics were used to summarize the patients’ age, sex, reporter occupation, and time-to-event in reports where CDK4/6 inhibitors were identified as the primary suspect. Age and time-to-event were summarized using the median and interquartile range (IQR), while categorical variables were summarized using counts and percentages. For some variables, missing or inappropriate values were indicated as unknown.

To identify signals for adverse events, the proportional reporting ratio (PRR), reporting odds ratio (ROR), and information component (IC) were used. The PRR and ROR are metrics widely used in the disproportionality analysis of spontaneous reports. A potential signal is identified when the number of adverse events is ≥3, the PRR and ROR are ≥2, and the chi-square is ≥4 [22,23]. The information component, based on the Bayesian Confidence Propagation Neural Network (BCPNN), considers a signal when the lower limit of the 95% confidence interval of the IC is ≥0 [24]. These metrics were used to identify drug and adverse event pairs that require further review. The formulas for the PRR, ROR, and IC are provided in Supplementary Table S2.

Median time-to-adverse event was evaluated for the selected drug and event pairs. Since not all reports contained valid information on the administration date of drugs and the date of the adverse event, time-to-event statistics could only be calculated for a subset of reports with valid date information [25]. Only reports with both the drug administration date and the adverse event date recorded in the 8-digit format were included. Reports were excluded if they contained illogical date information, such as a year beyond 2024 or a drug administration start date before 1980. Time-to-event was then analyzed for this refined subgroup.

### 2.4. Software

Python 3.7 was used for cleaning the raw data downloaded from the FAERS and for the overall statistical analysis. For visualization, Matplotlib 3.5.1 and Seaborn 0.11.2 were used.

### 2.5. Ethics Approval

The Institutional Review Board of the Jeonbuk National University granted an exemption for this study (IRB No. JBNU 2021-12-001).

## 3. Results

**Figure 1:**
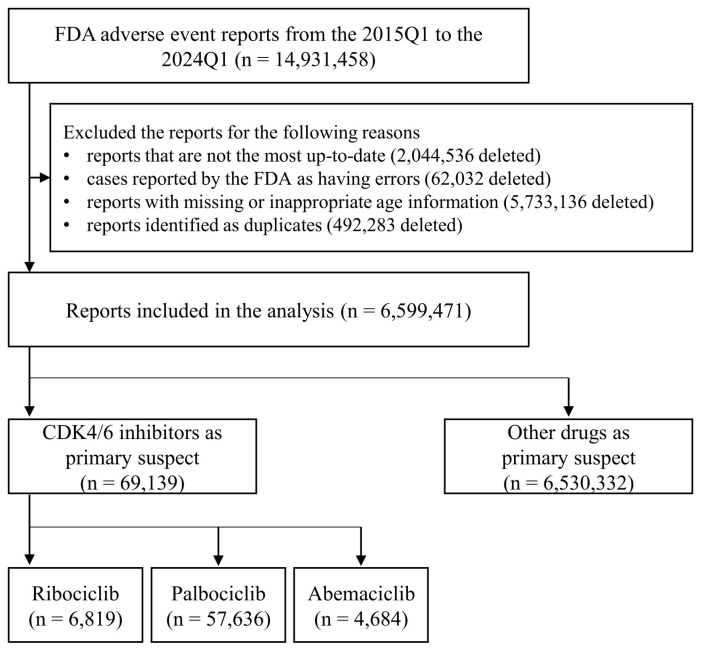
Flowchart of report selection. CDK4/6, cyclin-dependent kinase 4 and 6.

The flowchart for the selection of adverse event reports for analysis is presented in Figure 1. The number of reports included in the raw data file from 2015Q1 to 2024Q1, provided by the FDA Adverse Event Reporting System, was 14,931,458. Following the application of a series of filters, as detailed in the Methods Section, including the deduplication algorithms and the exclusion of cases with inappropriate age information, the number of retained reports was reduced to 6,599,471. Among them, 69,139 were identified as reporting CDK4/6 inhibitors as the primary suspect. Of these 69,139 reports, 6819 reported ribociclib, 57,636 reported palbociclib, and 4684 reported abemaciclib as the primary suspect.

Table 1 presents the characteristics of the 69,139 reports identifying CDK4/6 inhibitors as the primary suspect. The median age of the patients was 65 years (interquartile range [IQR] 57–74 years), and 96.6% (*n* = 66,783) of the reports were for female patients. The occupation of the reporter was healthcare professional in 41.9% (*n* = 28,939) of the cases, consumers in 44.6% (*n* = 30,832), and others in 13.5% (*n* = 9368). Time-to-event was calculated for the adverse event reports with valid drug administration start dates and dates when the adverse events occurred. In total, 26.6% (*n* = 18,370) of the reports had sufficient data to calculate the time-to-event. The median time-to-event was 69 days (IQR 18–260 days). Similar trends were observed when the overall reports were categorized by each drug for the various characteristics presented in Table 1. A total of 83.4% (*n* = 57,636) of the reports were related to palbociclib. When categorized by drug, the median age (IQR) was 66 years (IQR 57–74 years) for palbociclib, 61 years (IQR 50–71 years) for ribociclib, and 63 years (IQR 55–72 years) for abemaciclib. The majority of patients (over 95%) were female for all three drugs. Among the reports identifying the three CDK4/6 inhibitors as the primary suspect, the median time-to-event was 83 days (IQR 20–300 days) for palbociclib, 63 days (IQR 16–241 days) for ribociclib, and 33 days (IQR 11–109 days) for abemaciclib. Among the serious outcomes observed following the administration of CDK4/6 inhibitors, hospitalization (*n* = 11,728, 17%) and death (*n* = 9038, 13.1%) were the most common. Reports of life-threatening events, disability, and required interventions were relatively less frequent among the adverse event reports. This trend was consistent across reports for each of the CDK4/6 inhibitors.

A total of 2065 reports documented adverse events related to cardiac disorders (Table 2). When categorized by the three CDK4/6 inhibitors, there were 1304 reports for palbociclib, 598 for ribociclib, and 163 for abemaciclib. The most commonly reported cardiac adverse events following CDK4/6 inhibitor administration were myocardial infarction (*n* = 266, 12.9%), followed by cardiac disorder (*n* = 250, 12.1%) and atrial fibrillation (*n* = 234, 11.3%). Among the reports related to palbociclib, myocardial infarction was the most frequently reported (*n* = 194, 14.9%). For ribociclib, atrial fibrillation was most common (*n* = 67, 11.2%), and for abemaciclib, cardiac failure was the most frequently reported adverse event (*n* = 24, 14.7%).

The relationship between CDK4/6 inhibitors and cardiac adverse events at the SMQ level was explored using the PRR, ROR, and IC (Table 3). The pairs that included ribociclib and TdP/QT prolongation, and ribociclib and arrhythmia-related investigations, signs, and symptoms met the criteria of having ≥3 events, a PRR and ROR of ≥2, a chi-square value of ≥4, and an IC025 ≥ 0. Other pairs did not meet the pre-specified criteria for signal detection. Based on the number of reports, cardiac failure and hypertension were commonly reported across the three CDK4/6 inhibitors.

**Figure 2:**
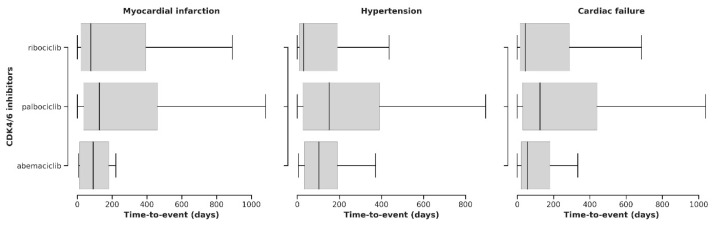
Median time-to-cardiovascular toxicity including TdP/QT prolongation, hypertension, and cardiac failure for CDK4/6 inhibitors. CDK4/6, cyclin-dependent kinase 4 and 6; TdP, torsade de pointes.

The SMQs of myocardial infarction, hypertension, and cardiac failure were selected for time-to-event analysis based on the number of reports. Figure 2 shows the median time-to-the development of myocardial infarction, hypertension, and cardiac failure for the three CDK4/6 inhibitors. Based on reports with available time-to-event statistics, the median time-to-event for myocardial infarction was 77 days (IQR 21–394 days) for ribociclib, 127 days (IQR 36–460 days) for palbociclib, and 91 days (IQR 13–181 days) for abemaciclib. For hypertension, the median time-to-event was 30 days (IQR 10–192 days) for ribociclib, 152 days (IQR 28–390 days) for palbociclib, and 103 days (IQR 34–191 days) for abemaciclib. For cardiac failure, the median time-to-event was 44 days (IQR 15–287 days) for ribociclib, 125 days (IQR 31–439 days) for palbociclib, and 56 days (IQR 22–182 days) for abemaciclib.

## 4. Discussion

The FAERS database is a valuable tool for safety signal identification, and its results can lead to FDA regulatory actions [26]. This study aimed to identify cardiovascular toxicity associated with the use of CDK4/6 inhibitors using the FAERS database. In addition to examining overall cardiovascular toxicity, this study focused on specific SMQs relevant to cardiovascular toxicity as classified in the consensus statement issued by the International Cardio-Oncology Society [20]. For the adverse event reports with ribociclib as the primary suspect, TdP/QT prolongation and arrhythmia-related investigations, signs, and symptoms were identified as meaningful adverse drug reaction signals.

Ribociclib has been known to prolong the QT interval based on previous clinical trial results [7]. The Warning and Precaution section of ribociclib’s labeling information also includes QT interval prolongation and includes guidance on dosage modification and relevant management when QT interval prolongation is confirmed by an electrocardiogram [27]. The identification of ribociclib and QT interval prolongation as a signal in this study corroborates that the methods and data used in this study are appropriate for identifying adverse events.

Among the analyzed cardiovascular toxicities, hypertension was the most frequently reported adverse event with CDK4/6 inhibitors as the primary suspect. A retrospective study using the OneFlorida Data Trust also found that incident hypertension was the most common cardiovascular adverse event associated with the use of CDK4/6 inhibitors [13]. In that study, the median time-to-the development of hypertension was 1.9 months, which is comparable to our findings of a median time-to-event of 30 days for ribociclib, 152 days for palbociclib, and 103 days for abemaciclib. Another study prospectively observed 12 patients treated with palbociclib and ribociclib and found an increase in blood pressure after six months compared to baseline [10]. This study also reported an increase in the arterial tissue-to-background ratio compared to baseline, suggesting that the observed hypertensive status might be induced by vascular inflammation [10]. Whether the changes in hypertension and related parameters were reversible was not determined in the study [10].

Heart failure was also a commonly reported adverse event in this study. Although heart failure was identified as the second most common cardiac adverse event after hypertension in a recent retrospective study [13], it has not been frequently reported as an adverse event associated with CDK4/6 inhibitors. Meanwhile, there has been a case report describing myocardial dysfunction and heart failure in a patient with metastatic breast cancer treated with abemaciclib and fulvestrant [11]. The patient’s ejection fraction recovered following the discontinuation of abemaciclib [11]. While the mechanism linking abemaciclib to heart failure is not well understood, an in vitro study has shown that abemaciclib inhibits cardiomyocyte viability [28]. Considering that the median time-to-the development of heart failure following CDK4/6 inhibitor administration is a few months, as observed in previous research and this study, it is warranted to carefully monitor signs and symptoms of heart failure during follow-up until more research findings and relevant management strategies are accumulated [13].

The higher number of cardiovascular events reported in post-marketing surveillance databases compared to clinical trials could be partially explained by the eligibility criteria of the clinical trials. One of the clinical trials that included palbociclib as an interventional drug excluded patients with a prolonged QTc interval, myocardial infarction, severe/unstable angina, arrhythmia, atrial fibrillation, symptomatic heart failure, or any cardiovascular conditions prior to trial participation [29]. Among the trials that tested the three CDK4/6 inhibitors, trials with ribociclib had the most restrictive eligibility criteria related to underlying cardiovascular status. Patients with active cardiac disease or various types of cardiac dysfunction, including angina pectoris, pericarditis, myocardial infarction, heart failure, and cardiac arrhythmia, were all excluded [7]. Additionally, patients were excluded if vital signs showed a heart rate < 50 beats/min or >90 beats/min, or systolic blood pressure > 160 mmHg or <90 mmHg [7]. In contrast, the MONARCH-3 clinical trial for abemaciclib had the most lenient cardiovascular-related eligibility criteria, excluding only those with syncope of cardiovascular etiology, ventricular tachycardia, ventricular fibrillation, or sudden cardiac arrest [30]. A retrospective study by Fradley et al. observed a higher incidence of cardiovascular adverse events in patients with cardiovascular disease-related risk factors such as diabetes, hyperlipidemia, and hypertension [13]. The exclusion of patients with cardiovascular disease in past clinical trials could be a reason why different cardiovascular toxicity profiles are observed in real-world settings.

According to the analysis in this study, adverse events following CDK4/6 inhibitor use occurred at a median of 69 days. When focused on specific cardiovascular adverse events such as myocardial infarction, hypertension, and cardiac failure, the median onset was longer, typically extending by a few months. Cardiovascular toxicity following CDK4/6 inhibitor treatment has not received sufficient attention, with the exception of QTc interval prolongation. Previous studies have also shown that cardiovascular toxicity is more common in patients with risk factors such as diabetes and hyperlipidemia [13]. Therefore, it is important to monitor cardiovascular function more closely in patients who have these risk factors.

This study has a few limitations. Firstly, because the FAERS database collects spontaneous reports, it does not necessarily represent the complete safety profile for each drug. Secondly, not all information in the FAERS database is accurately recorded. During the data cleaning process for analysis, information such as age, event date, and drug administration start date was assessed for validity, which led to the exclusion of a substantial number of reports. Thirdly, detailed information on the patients’ past medical history or laboratory measurement results is not available. Information on whether the patients had pre-existing risk factors for cardiovascular disease, such as heart failure or QT prolongation, as well as other factors like smoking status or body mass index, is not available. Also, the dataset did not specify the exact methodologies used to diagnose the adverse events. Fourthly, the signals identified in the FAERS do not imply causality. Therefore, additional research is needed to understand the mechanisms underlying the observed cardiovascular toxicity.

However, this study has several strengths. Unlike previous studies that reported on cardiovascular toxicity with limited scope, often involving only a few case reports or small groups of patients, this study provides a comprehensive analysis of cardiac disorders and cardiovascular toxicity for all currently available CDK4/6 inhibitors. Additionally, this study offers insights into the median time-to-onset of adverse events following the administration of CDK4/6 inhibitors by providing analysis results for the subgroup of reports that included time-to-event information. Specifically, the median time-to-event for cardiac failure and hypertension, which had a high number of cases, extended up to approximately 200 days. Finally, by providing reporting frequencies and signal detection analysis results using the PRR, ROR, and IC025, this study helps prioritize which cardiovascular toxicities warrant monitoring in real-world clinical settings and identifies areas where further research is needed.

## 5. Conclusions

This study analyzed adverse reactions associated with the use of CDK4/6 inhibitors using the FAERS database. QT prolongation and arrhythmia with ribociclib use were identified as signals, corroborating the information already included in the drug’s label. Additionally, high reporting frequencies of hypertension and cardiac failure were observed, indicating the need for careful monitoring when using CDK4/6 inhibitors.

## Figures and Tables

**Table 1:** The characteristics of the FDA adverse event reports with CDK4/6 inhibitors as the primary suspect.

Characteristics	CDK4/6 Inhibitors	Palbociclib	Ribociclib	Abemaciclib
Total	69,139	57,636	6819	4684
Age (years)				
Median (IQR)	65 (57–74)	66 (57–74)	61 (50–71)	63 (55–72)
Sex				
Female	66,783 (96.6)	55,757 (96.7)	6518 (95.6)	4508 (96.2)
Male	1749 (2.5)	1491 (2.6)	150 (2.2)	108 (2.3)
Unknown	607 (0.9)	388 (0.7)	151 (2.2)	68 (1.5)
Reporter occupation				
Healthcare professional	28,939 (41.9)	24,424 (42.4)	2905 (42.6)	1610 (34.4)
Consumer	30,832 (44.6)	25,349 (44.0)	3316 (48.6)	2167 (46.3)
Other	9368 (13.5)	7863 (13.6)	598 (8.8)	907 (19.4)
Time-to-event (days)				
No. of reports	18,370 (26.6)	12,701 (22.0)	3623 (53.1)	2046 (43.7)
Median (IQR)	69 (18–260)	83 (20–300)	63 (16–241)	33 (11–109)
Serious outcomes				
Death	9038 (13.1)	6770 (11.7)	1815 (26.6)	453 (9.7)
Life-threatening	729 (1.1)	378 (0.7)	250 (3.7)	101 (2.2)
Hospitalization	11,728 (17)	8470 (14.7)	1978 (29)	1206 (25.7)
Disability	339 (0.5)	201 (0.3)	91 (1.3)	47 (1)
Required intervention	107 (0.2)	70 (0.1)	16 (0.2)	21 (0.4)

CDK4/6, cyclin-dependent kinase 4 and 6; IQR, interquartile range.

**Table 2:** Most frequently reported cardiac adverse events identifying CDK4/6 inhibitors as primary suspect.

Characteristics	Total	Palbociclib	Ribociclib	Abemaciclib
Total number of reports	2065	1304	598	163
Top 5 Preferred Terms, *n* (%)				
1	Myocardial infarction, 266 (12.9)	Myocardial infarction, 194 (14.9)	Atrial fibrillation, 67 (11.2)	Cardiac failure, 24 (14.7)
2	Cardiac disorder, 250 (12.1)	Cardiac disorder, 192 (14.7)	Myocardial infarction, 58 (9.7)	Atrial fibrillation, 21 (12.9)
3	Atrial fibrillation, 234 (11.3)	Palpitations, 150 (11.5)	Arrhythmia, 55 (9.2)	Tachycardia, 18 (11.0)
4	Palpitations, 208 (10.1)	Atrial fibrillation, 146 (11.2)	Tachycardia, 50 (8.4)	Palpitations, 17 (10.4)
5	Cardiac failure, 175 (8.5)	Cardiac failure, 108 (8.3)	Cardiac disorder, 46 (7.7)	Myocardial infarction, 14 (8.6)

**Table 3:** Number of reports of cardiotoxicities and signal profiles.

Drug	SMQ	N	PRR	ROR	Chi-Square	IC	IC025
Ribociclib	TdP/QT prolongation	190	8.43	8.65	1237.99	3.07	2.86
	Arrhythmia-related investigations, signs, and symptoms	11	8.19	8.2	68.83	3.15	2.33
	Cardiac failure	166	1.8	1.82	60.04	0.86	0.64
	Hypertension	164	1.08	1.08	0.9	0.11	−0.11
	Cardiomyopathy	19	0.99	0.99	0	0.06	−0.57
	Noninfectious myocarditis/pericarditis	4	0.34	0.34	5.25	−1.25	−2.52
	Myocardial infarction	79	1.12	1.12	1.08	0.19	−0.13
Palbociclib	TdP/QT prolongation	31	0.16	0.16	136.57	−2.58	−3.08
	Arrhythmia-related investigations, signs, and symptoms	0	-	-	11.54	−3.52	−6.35
	Cardiac failure	587	0.75	0.75	48.36	−0.41	−0.52
	Hypertension	766	0.59	0.59	218.41	−0.75	−0.85
	Cardiomyopathy	43	0.26	0.26	88.52	−1.88	−2.31
	Noninfectious myocarditis/pericarditis	6	0.06	0.06	89.83	−3.84	−4.91
	Myocardial infarction	224	0.37	0.37	235.30	−1.40	−1.59
Abemaciclib	TdP/QT prolongation	1	0.06	0.06	13.72	−2.96	−4.96
	Arrhythmia-related investigations, signs, and symptoms	0	-	-	0.93	0.1	−2.73
	Cardiac failure	53	0.84	0.84	1.7	−0.23	−0.62
	Hypertension	16	0.15	0.15	76.91	−2.62	−3.31
	Cardiomyopathy	10	0.76	0.76	0.77	−0.26	−1.11
	Noninfectious myocarditis/pericarditis	2	0.24	0.24	4.67	−1.45	−3.08
	Myocardial infarction	23	0.48	0.47	13.41	−1.01	−1.59

SMQ, Standardized MedDRA Queries; PRR, proportional reporting ratio; ROR, reporting odds ratio; IC, information component; IC025, lower limit of 95% confidence interval for IC; TdP, torsade de pointes.

## Data Availability

The FDA Adverse Event dataset is available through the FDA Adverse Event Reporting System. The scripts for the analysis are available from the following GitHub repository: https://github.com/kimkimjh/cdk-cardio (accessed on 23 July 2024).

## Supplementary Materials

The following supporting information can be downloaded at https://www.mdpi.com/article/10.3390/cancers16162869/s1, Table S1: The list of Preferred Terms that constitute the Standardized MedDRA Queries. Table S2: The formulas for the PRR, ROR, and IC.
