# Supplementary material for: Profiling the Cardiovascular Toxicities of CDK4/6 Inhibitors: A Real-World Pharmacovigilance Study
Source: Cancers (Basel). 2024 Aug 17;16(16):2869. doi: 10.3390/cancers16162869 (PMC11352810; doi:10.3390/cancers16162869)
Supplement: Supplementary file 1 [file cancers-16-02869-s001.zip › supplementary_S2.pdf]

**Supplementary Table S2.** The formulas for PRR, ROR, and IC.

| Measure | Formula |
|---|---|
| PRR | $\frac{\frac{n_{11}}{n_{11}+n_{12}}}{\frac{n_{21}}{n_{21}+n_{22}}}$ |
| ROR | $\frac{\frac{n_{11}}{n_{12}}}{\frac{n_{21}}{n_{22}}}$ |
| IC | $\frac{1}{ln(2)} \times \left[ln\left(\frac{1+n_{11}}{4+n}\right) - ln\left(\frac{1+n_{11}+n_{12}}{2+n}\right) - ln\left(\frac{1+n_{11}+n_{21}}{2+n}\right)\right]$ |

$n_{11}$, The number of cases where both the drug of interest and the adverse event of interest are reported together.

$n_{12}$, The number of cases where the drug of interest is reported without the adverse event of interest.

$n_{21}$, The number of cases where the adverse event of interest is reported without the drug of interest.

$n_{22}$, The number of cases where neither the drug of interest nor the adverse event of interest are reported.

$n$, The total number of cases in the dataset,

PRR, proportional reporting ratio; ROR, reporting odds ratio; IC, information component
